# The return of Pfeiffer’s bacillus: Rising incidence of ampicillin resistance in *Haemophilus influenzae*

**DOI:** 10.1099/mgen.0.000214

**Published:** 2018-09-12

**Authors:** Eva Heinz

**Affiliations:** Wellcome Trust Sanger Institute, Cambridge CB10 1SA, UK

**Keywords:** *Haemophilus influenzae*, emerging pathogen, ampicillin resistance, Hib vaccine

## Abstract

*Haemophilus influenzae,* originally named Pfeiffer’s bacillus after its discoverer Richard Pfeiffer in 1892, was a major risk for global health at the beginning of the 20th century, causing childhood pneumonia and invasive disease as well as otitis media and other upper respiratory tract infections. The implementation of the Hib vaccine, targeting the major capsule type of *H. influenzae*, almost eradicated the disease in countries that adapted the vaccination scheme. However, a rising number of infections are caused by non-typeable *H. influenzae* (NTHi), which has no capsule and against which the vaccine therefore provides no protection, as well as other serotypes equally not recognised by the vaccine. The first line of treatment is ampicillin, but there is a steady rise in ampicillin resistance. This is both through acquired as well as intrinsic mechanisms, and is cause for serious concern and the need for more surveillance. There are also increasing reports of new modifications of the intrinsic ampicillin-resistance mechanism leading to resistance against cephalosporins and carbapenems, the last line of well-tolerated drugs, and ampicillin-resistant *H. influenzae* was included in the recently released priority list of antibiotic-resistant bacteria by the WHO. This review provides an overview of ampicillin resistance prevalence and mechanisms in the context of our current knowledge about population dynamics of *H. influenzae*.

## Data Summary

**Fig. 1.**
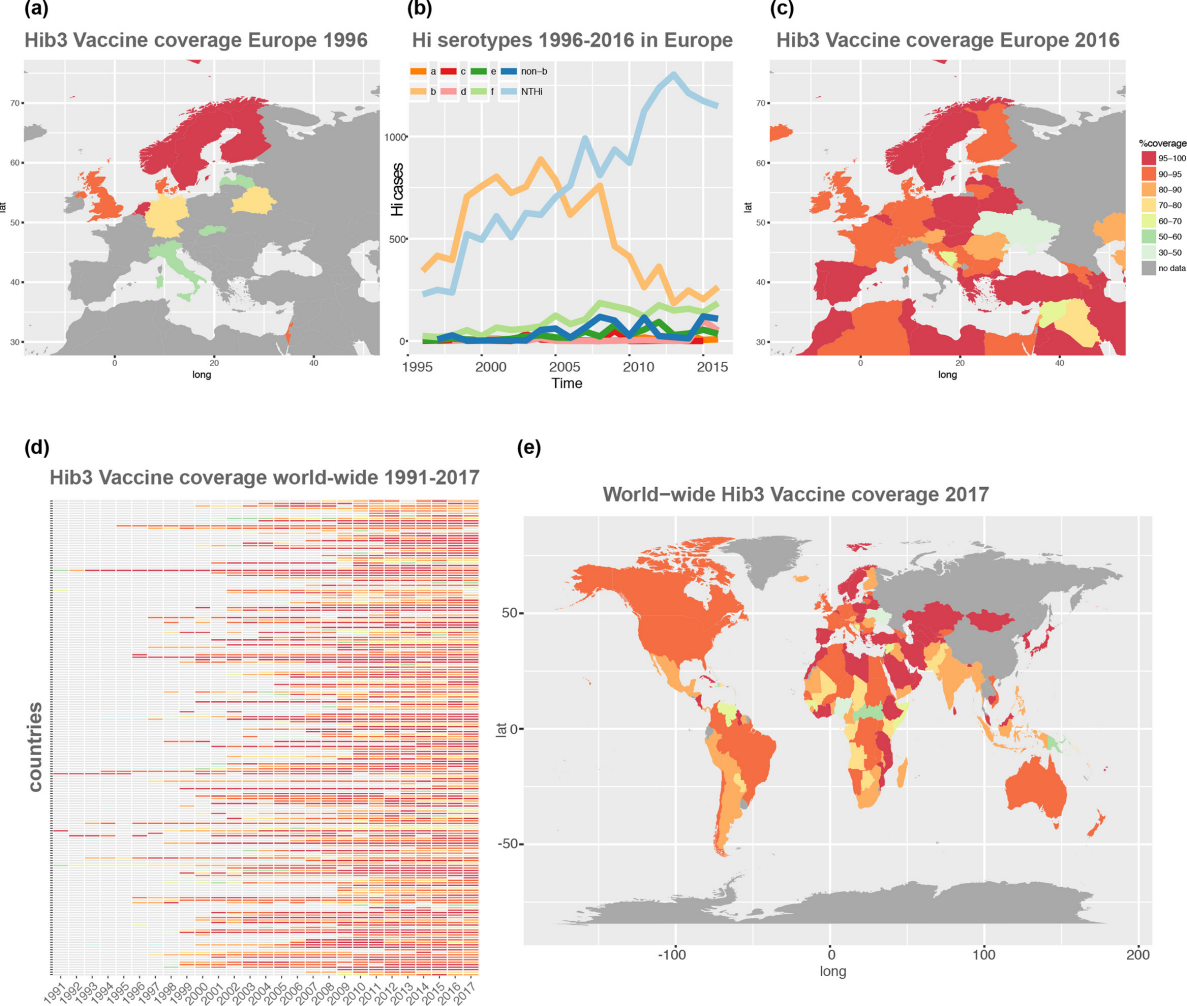
Drastic change in *H. influenzae* population following Hib vaccination. (a) The coverage of Hib vaccination 1996 shows low only very sparse coverage. (b) The change in serotypes combining data from all European countries as retrieved from the European Centre for Disease, ranging from 1996 with predominantly serotype B to 2016, where the population consists almost entirely of NTHi strains. (c) The vaccination coverage for Europe in 2016, showing very high vaccination coverage. (d) The introduction of Hib vaccine globally from 1991 until 2017. (e) The global vaccine coverage 2017 is almost complete and it can be speculated that the *H. influenzae* population equally consists mainly of NTHi strains, as is also shown by isolated studies, although global surveillance data of serotype prevalance compared to the European data is not available.

**Fig. 2.**
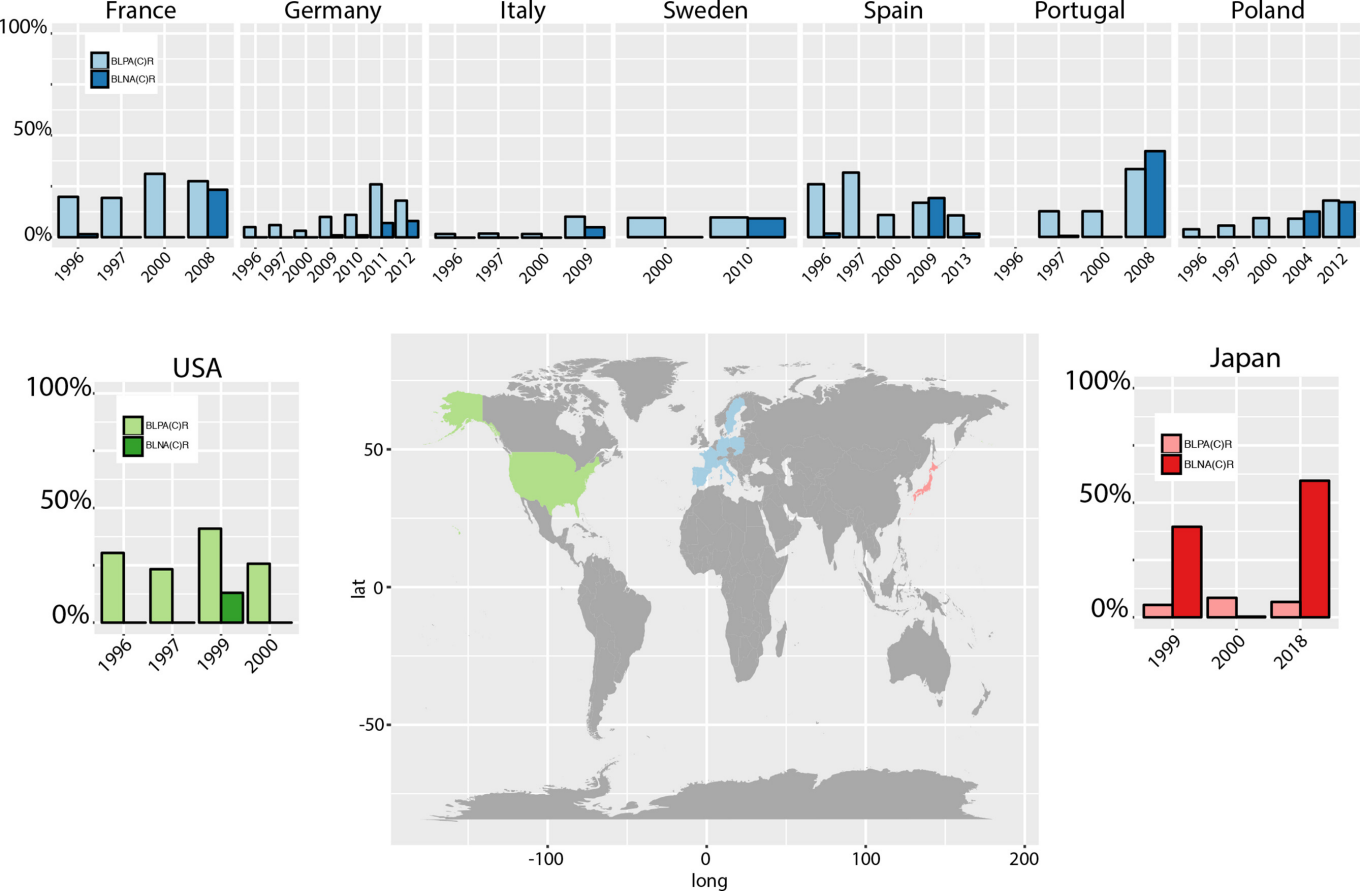
Global increase in BLPAR and BLNAR resistance. Data shown was summarised from [47, 50, 52, 53, 55, 106–113]; BLNA(C)R and BLPA(C)R also include the respective strains with cephalosporin (amoxicillin/clavulanate) resistance. The included studies assessed exclusively or to the vast majority NTHi isolates, and are recent surveillance studies including the reporting of different resistance mechanisms (BLNAR, BLPAR) shown here as bar charts. For studies summarising isolates over several years, the last year of the sample origins in the study is used for the order along the x-axis.

The data for Fig. 1 for different serotypes per country is derived from the ECDC (https://ecdc.europa.eu/en/invasive-haemophilus-influenzae-disease/atlas), the data for the Hib vaccine implementation from the WHO (http://www.who.int/immunization/monitoring_surveillance/data/en/). Fig. 2 shows a summary of several studies, all data is available from the previously published studies and reports, and is clearly cited in the text and listed in the bibliography.

**Impact Statement**

*Haemophilus influenzae* is a human pathogen and causes respiratory disease as well as invasive disease, such as sepsis/bacteraemia and meningitis, and was a main cause of childhood mortality at the beginning of the 20th century. The introduction of the Hib vaccine, targeting the main serotype b, almost eradicated Hib disease from countries which have implemented it in their vaccination scheme. With the disappearance of Hib however, a strong increase followed in disease caused by non-typeable *H. influenzae*, which is now the leading disease-causing group. There is a steady rise in antimicrobial resistances, in particular ampicillin resistance, and an increasing number of reports of additional resistance against macrolides, fluoroquinolones and other beta-lactams, including carbapenems. This review highlights the continuous rise of resistance in the *H. influenzae* population, which is strongly driven through changes in the chromosome leading to intrinsic resistance. Intrinsic mechanisms are often more complex and less well-understood than resistance through acquired genes, and there is an urgent need for more surveillance and population studies of *H. influenzae*.

## Introduction

*H. influenzae* is a diverse species and has traditionally been classified by its polysaccharide capsule, which can either be entirely absent (non-typeable *H. influenza*, NTHi [1]), or one of six serotypes (a–f; [1–3]). In the 1980s, one of the most successful vaccines to date, the Hib vaccine, was widely implemented against the major disease-causing form of *H. influenzae*, serotype b; this serovar almost disappeared in countries which introduced Hib into their vaccination scheme [4] (Fig. 1a–c). The introduction of the vaccine in 73 countries supported by the Global Alliance for Vaccines and Immunizations (GAVI) was estimated to save 1.4–1.7 million lives 2011–2020 [5], and was introduced into all GAVI-supported countries by 2014 [6] (Fig. 1d, e). The success of the Hib vaccine is accompanied by rising numbers of NTHi as well as, to a much lesser extent, other serotypes [7–9], which fill the niche left by the strongly reduced prevalence of *H. influenzae* b [10–13] (Fig. 1b). In post-Hib settings NTHi is now the main pathogenic lineage, causing upper respiratory tract and ear infections and chronic obstructive pulmonary disease (COPD) infections [14, 15] which is the third leading cause of death world-wide [16], and is one of the three leading causes of acute otitis media (AOM; together with *Streptococcus pneumoniae* and *Moraxella catarrhalis* [17]). NTHi is also the main type causing invasive disease, including sepsis and meningitis [11, 12, 17–19]. Even though invasive disease, especially meningitis, was mostly driven by Hib in the pre- and sometimes also the post-vaccination period [20, 21], several reports show NTHi meningitis cases comprise up to 69 % of the *H. influenzae*-derived meningitis [19, 22–25]. For detailed discussion of virulence and interaction with the host the readers are pointed to several recent expert reviews [10–13, 17, 26], these aspects will not be covered in detail here.

The main treatment against *H. influenzae* is ampicillin, a member of the beta-lactams. These interfere with the biosynthesis of the Gram-negative cell wall through binding of the enzymes which cross-link the peptidoglycan subunits, the penicillin-binding proteins (PBPs [27, 28]). Key resistance mechanisms are either acquired enzymes that hydrolyse beta-lactams (beta-lactamases), or target modifications through changes in the PBP sequences. The majority of high-risk multi-drug-resistant Gram-negative pathogens to date acquired beta-lactamases, which mainly spread through mobile elements [29, 30]. There has been a steady increase in *H. influenzae* isolates resistant to ampicillin, including a high number of intrinsic resistance cases, and tracking of the epidemiology is challenging due to the genomic diversity of *H. influenzae* and its high prevalence of recombination. The importance of an increasing level of resistance amongst *H. influenzae* isolates has been recognised by the WHO, and it was included in the recently released list of high-priority antimicrobial-resistant pathogens [31]. This review will profile this currently understudied pathogen with an emphasis on the increasing prevalence of ampicillin resistance and the rising number of resistance mechanisms against other antimicrobial classes in the context of its complex population.

### Plasmid-derived ampicillin resistance

Although the first report of resistance occurred in 1972 [32], *H. influenzae* is still largely treatable by most major groups of antimicrobials. The first beta-lactam-resistance mechanisms observed relied on the acquisition of the beta-lactamases *blaTEM-1/2* and *blaROB-1* [33, 34], and *blaTEM-1* is the by far more prevalent gene. It moved via the associated Tn2/3 transposon onto a cryptic plasmid already present in *H. influenzae*, which can also integrate into the chromosome and carries several resistances [18, 35]. More recently distribution of mobile resistances was also described on small but conjugative plasmids, and transfer to *Escherichia coli*, as shown *in vitro*, indicates the possibility of inter-species resistance spread [36]; others of these small plasmids lack the conjugation machinery. However, other common *H. influenzae* plasmids were highly unstable when transformed into *E. coli* [37]; possibly of higher relevance as plasmid reservoirs for *H. influenzae* are closely related species like *Pasteurella multocida*, *Haemophilus haemolyticus* and *Haemophilus parasuis* [35, 37, 38]. Studies have shown the presence of conjugative plasmids with *bla*ROB-1 shared amongst these species; however, these plasmids have a significant impact on the fitness of *H. influenzae*, which might explain the low prevalence of *bla*ROB-1 [37]. However, due to the highly permeable cell envelope of *H. influenzae* [33, 39], there is an increased influx of beta-lactams [39]. The otherwise widespread *blaTEM-1* enzyme thus needs to be modified to confer resistance in *H. influenza*, either through modification of the promoter to achieve higher expression or through modification of the enzyme itself [40–43].

### Intrinsic resistance against ampicillin and other beta-lactams

*H. influenzae* encodes four PBPs, however several studies have shown that there is no correlation between resistances and mutations in PBPs 1, 2 and 4 [44, 45]. PBP3 however, encoded by the gene *ftsI*, clearly confers resistance against ampicillin, and includes several mutations around functional areas of the protein. Ubukata *et al*. [46] initiated a typing scheme which can be used to classify PBP3 according to its mutations, and currently comprises four classes [47, 48]. The main resistance-conferring mutations are in one of the two key functional sites, disrupting the Lys–Thr–Gly (KTG) or the Ser–Ser–Asn (SSN) motif, which lead to a decrease in binding of ampicillin to PBP3 [45–47]. The thus intrinsically resistant strains against ampicillin are referred to as BLNAR, or beta-lactamase-negative ampicillin-resistant (Fig. 2); BLNAR strains that acquired a beta-lactamase in addition are referred to as beta-lactamase-positive amoxicillin-clavulanate resistant (BLPACR), however this group is usually only found at low prevalence. Whilst initially BLNAR was mainly observed in Japan, where Hib was only introduced in 2009 to the vaccination scheme [49], BLNAR has spread across the globe in the past 20 years (Fig. 2). NTHi, the, to date, most prevalent *H. influenzae* type causing disease, is often associated with considerable proportions of resistance in recent studies [50], with similar rates of acquired and intrinsic (BLNAR) resistance mechanisms [51–54]. BLNAR strains are grouped, according to their resistances, into low-level (MIC 1 µg ml^−1^; sensitive=MIC 0.25 µg ml^−1^) and high-level (MIC 2 µg ml^−1^) resistance. High-level-resistant strains also show resistance against cephalosporins [46, 47] and have increased in prevalence in parts of Asia [48, 55–59]. In addition, upregulation of AcrAB, combined with PBP3 changes, can lead to further resistance to cephalosporins as well as carbapenems, the last-line drugs that are widely applicable against Gram-negative infections [47, 60–64].

### Rising incidence of resistances against other drugs through intrinsic mechanisms

Given the increasing trend in resistances, the treatment options for *H. influenzae* are becoming increasingly limited. Azithromycin, a macrolide, is widely prescribed in respiratory disease, although there is need for caution against over-use to prevent selection for resistance [65]. Similar to ampicillin resistance, *H. influenzae* modifies the macrolide targets (50S ribosomal RNA and ribosome-binding proteins), leading to high levels of resistance [66, 67]. This mechanism is in addition to the ubiquitously present AcrAB multidrug-efflux pump which provides a baseline reduced susceptibility, and the authors of recent studies have reported changes in the AcrAB sequence to further contribute to resistance [68]. Another major alternative are quinolones, however, recently there has been an accumulation of reports of fluoroquinolone resistance [69–72] again through mutations in the targets, DNA gyrase (encoded by *gyrA* and *gyrB*) and topoisomerase IV (encoded by *parC* and *parE*); and thus again conferring resistance through a plasmid-independent mechanism, making it easy for resistances to arise independently through point mutations and/or spread through recombination.

### *H. influenzae* population structure

Not only recognised as a pathogen very early on, *H. influenzae* was also the bacterium that opened the area of whole-genome sequencing, representing the first complete genome from a free-living organism sequenced (in 1995) [73]. One of the main challenges of NTHi is its great diversity; whilst the capsulated lineages are mainly clonal expansions [74], with each serotype presenting a monophyletic lineage, initial multi-locus sequence typing (MLST) studies already recognised the increased recombination, and as a consequence higher diversity, of NTHi [75]; and in population studies, there are often almost as many STs in a dataset as NTHi sequences [76–78]. NTHi can also persist in a host over several years, which not only leads to changes in virulence genes as well as potential vaccine targets [79, 80], but also increases its exposure to antimicrobials to treat unrelated infections of the host, and is another risk factor in the acquisition or spread of resistances through point mutations and recombination, respectively. Recombination in *H. influenzae* is elevated for the whole species through its natural competence, a bacterial mechanism increasing the uptake of DNA from the same species. This is achieved by a DNA uptake machinery located at the cell envelope that shows a strong bias toward certain short DNA sequence motifs, and the genomes of the respective bacteria are highly enriched in the preferred sequences, mostly equally distributed throughout the genome and also reflected in the encoded peptides [81–83]. This leads to an increased ability to recombine between different strains [78, 84], and thus also facilitates easy exchange of chromosomal resistance mechanisms, for example *ftsI* [85]. In addition, recombination between related species has been observed, and leads to spread of resistance through exchange of the chromosomally encoded target gene *ftsI* [86].

### Interactions with other species

*H. influenzae* shares its niche with *Streptococcus pneumonia*e and *Moraxella catarrhalis* in the nasopharynx, three of the four primary pathogens (with *Pseudomonas aeruginosa*) causing COPD [87], and the three leading pathogens of AOM [17]. Similar to the success of the Hib vaccine, the main disease-causing serovars of *S. pneumoniae* were successfully controlled with several vaccines, but also here we see an increase in serovars not targeted by the available vaccines and in and drug resistances, including penicillin as well as macrolides [88–91]. Mixed infections further provide the potential to share resistance mechanisms across species without actual gene transfer: *Moraxella catarrhalis* can secrete outer membrane vesicles (OMVs) containing beta-lactamase which hydrolyse beta-lactams, and thus protect *de facto* sensitive *S. pneuomoniae* and *H. influenzae* strains from the antimicrobial [92], as well as complement resistance factors, protecting from the immune system [87]. A similar effect was observed with beta-lactamase-containing OMVs secreted by *H. influenzae* protecting group A Streptococci, another common component of the nasopharynx microbiota [93]. More studies for a better understanding are also required to understand the impact of the widely used pneumococcal vaccines. Results from a recent study have indicated that introduction of the *S. pneumoniae* vaccine resulted in increased carriage of NTHi, although not due to the expansion of a particular lineage/sequence type [94]. This study is, however, in contrast to other reports, where a reduction of NTHi carriage following vaccination with pneumococcal vaccine was observed [95–98]. Other studies reported no change in NTHi following pneumococcal vaccination; given the different study designs and the differences in pneumococcal vaccines, some of which include the *H. influenzae* protein D as carrier, more data will be required to better understand the impact of different pneumococcal vaccines on *H. influenzae* [17, 99, 100].

### Conclusion

*H. influenzae* is a major pathogen, considering respiratory as well as invasive disease. The success of the Hib vaccine has been followed by a global increase of NTHi, which receives little attention from public media and is not included in major resistance surveillance programs. This is likely to be since NTHi can still readily be treated in most cases, but there is an alarming rise in intrinsic resistances against beta-lactams, as well as other classes. While a lot of focus is placed on acquired resistance mechanisms, the epidemiology of intrinsic resistance is much less understood; this is additionally challenging for NTHi, which has a highly diverse population structure and can undergo recombination, including exchange of resistance determinants, within the species as well as with related species. Intrinsic resistance, especially against beta-lactams, is also a significant problem for future drug developments. A major effort to control the current crisis in antimicrobial resistance is the development of beta-lactamase and carbapenemase inhibitors [101–103] used in combination with beta-lactams and carbapenems, respectively, to revert cells with these acquired enzymes to be *de facto* susceptible. There are currently 10 inhibitors in clinical development, but changes in PBP3 and AcrAB escape these treatment options [104]. Given the limited amount of genomic surveillance data, and that *H. influenzae* resistance is not included in current antimicrobial-resistance monitoring programs [105], the extent of the increase in resistant isolates, as well as the population dynamics leading to the spread of resistance, are not clear. There is an urgent need for better surveillance of resistant *H. influenzae*, understanding of the population dynamics, including recombination events, and the influence of pneumococcal vaccines.
